# "Passive victim – strong survivor"? Perceived meaning of labels applied to women who were raped

**DOI:** 10.1371/journal.pone.0177550

**Published:** 2017-05-11

**Authors:** Michael Papendick, Gerd Bohner

**Affiliations:** Department of Psychology, University of Bielefeld, Bielefeld, Germany; Goethe-Universitat Frankfurt am Main, GERMANY

## Abstract

Three experiments (total *N* = 464) were conducted in parallel with English- and German-speaking participants to examine the perceived meanings and effects of the labels "victim" versus "survivor" (and their German equivalents) when applied to a woman who was raped. In Study 1 (*N* = 179), participants read a rape vignette and then rated the meaning of the label it contained (either "victim" or "survivor") on a 15-item semantic differential. Independent of language and participant gender, "survivor" was perceived more positively overall (e.g., as *strong*, *brave*, *active*) than was "victim" (*weak*, *passive*, but also *innocent*). In Study 2 (*N* = 95), labels were varied within items assessing judgments of an acquaintance-rape case (e.g., *"Does the victim [survivor]* … *carry a certain responsibility for what happened*?*"*), focusing on short-term outcomes. Significant interaction effects of label and participants’ gender emerged on case-related judgments. Participants in both language samples judged "survivor" to be a less appropriate term than "victim". In Study 3 (*N* = 190), participants read a text in which a woman who had been raped labeled *herself* as either "victim" or "survivor", focusing on the coping with sexual violence. As in Study 2, German-language participants showed no significant effects of the label on their case judgments but rejected the term "survivor" as inappropriate; English-language participants, by contrast, perceived the woman describing herself as "survivor" to be more psychologically stable and regarded the use of both labels as appropriate. Results are discussed in terms of their applied relevance for communicating about sexual violence.

## Introduction

Perceptions of sexual aggression include various aspects and are influenced by a host of factors. Research in this area, mostly quantitative studies presenting rape vignettes with varying content, has looked at several dependent variables, including social perceptions of perpetrators and victims, judgments of severity and guilt, and attributions of blame. Independent variables that were addressed include characteristics of victims, perpetrators, and observers as well as situational variables. For instance, observers tend to perceive an incident as rape and blame the victim less if the victim showed some resistance [1], but blame the victim more in the case of an acquaintance rape than a stranger rape [2]. On the observer side, strong predictors are participant's gender, with male participants blaming the victim more [3, 4], acceptance of rape myths [5, 6], and sexist attitudes [2]. In sum, prior studies underline that the perceptions of sexual violence are influenced by features of the situation as well as observer, victim, and perpetrator characteristics and their interactions (for extensive reviews, see [7, 8, 9]).

Another potentially influential factor that has rarely been taken into account so far is the label used to refer to the person who was raped. A person who was raped is commonly referred to as the "victim", but another label that appears to be increasingly popular is the term "survivor". In the studies reported here we will examine the meanings that observers ascribe to these labels and explore the potential effects that these labels may have on observers' perceptions of rape cases and of the persons thus labeled. We will do so by comparing perceptions of experimental case vignettes that differ only in the label presented but are identical in all other respects. Although it may appear unlikely that such a purely linguistic variation should have much of an effect on case-related judgments, existing research on linguistic factors suggests otherwise. For example, Bohner [10] asked participants to describe in writing a film scene of an acquaintance rape that they had watched. He found that participants high (vs. low) in rape myth acceptance, who were motivated to exonerate the perpetrator, used more passive voice and nominalizations to describe the perpetrator's actions (e.g., "and then she was raped"; "the rape occurred"), thus shifting the focus of attention on the victim or at least away from the perpetrator. Furthermore, participants' use of the passive voice was positively correlated with their judgments of victim blame. Similar evidence for "linguistic avoidance" of mentioning the perpetrators was reported by Henley, Miller, and Beazley [11], who found that American newspapers used the verb form "raped" much more frequently in the passive (70%) than active (30%) voice, whereas passives were much less frequent for positive verbs (e.g. "thanked") or neutral verbs (e.g., "touched"). Henley and her colleagues interpreted these results as indicating a tendency of writers to place rapists' responsibility in the background (for related evidence from texts on men battering women, see [12]).

If seemingly equivalent verb forms suggest different degrees of blame, then different labels used to designate women who were raped might also affect perceptions of rape. In everyday communication about sexual violence we frequently use the socially established term of the rape victim, the person who falls victim to the perpetrator. Using this term has become so natural that speakers and readers may mostly be unaware of its potential implications. Yet the term itself may evoke associations influencing our impression of the person it refers to, such as imagining him or her as someone with a weak physical constitution who passively suffers from severe long-term consequences of the experienced violence. Persons who have been raped nowadays may reject the term's use because of its negative associations, whereas they are likely to prefer a self-description as a "survivor", associated with connotations of activity, strength, optimism, and positive coping with a potentially life-threatening experience [13].

As we are going to study the labels "victim" and "survivor" with English-speaking participants, and their equivalents "Opfer" and "Überlebende" with German-speaking participants, we first consider these terms' common definitions. Both the English term "victim" [14] and the German term "Opfer" [15] provide a variety of potential interpretations. While both can imply an injury as well as a certain probability to die when referring to a violent crime, an accident or a natural disaster, they can also be applied to nonviolent acts, as being tricked or becoming the victim of mobbing or verbal abuse. Likewise the English term "survivor" and the German term "Überlebende" can imply that an event experienced has been very severe and potentially life-threatening. In the context of sexual violence the terms may stress the physical invasiveness of the event and point to a coexistent threat to life above and beyond the sexual nature of the assault. At the same time both terms comprise the important aspect of continuing to live subsequent to negative experiences. In the context of sexual violence this metaphorical use of the term may play an important role in the long-term outcomes and the process of coping with negative experiences, regardless of whether the event itself has been threatening to life or not. In modern language especially the English term "survivor" and its corresponding verb "survive" are frequently used in non-lethal contexts, as in "surviving divorce" or "surviving high school", emphasizing that someone does "live on" after experiencing a stressful but not life-threatening event. Depending on whether an event’s immediate, physical outcomes or a person’s subsequent processing of the experience are emphasized the terms "survivor" and "Überlebende" may carry different meanings.

Beyond that commonality, meaningful differences exist between each of the two language versions. In German youth language, the term "Opfer" is commonly used to refer to someone who is regarded a weakling or loser. The English "survivor" is more distinctly defined as a person who continues to live despite an invasive experience, whereas the definition of the German "Überlebende" focuses more on merely staying alive and appears less future-oriented and forward-looking.

The use of both labels in the context of sexual violence is discussed extensively, especially in feminist discourse. Common connotations associated with "victim" in theoretical works are unawareness, helplessness, and especially passivity [16]. A potential origin of this negatively connoted image is the discourse on domestic violence against women that started in the 1970s [17]. The lack of public attention to this kind of violence and even charges brought against women, justifying the violence as the result of women's alleged maladaptation, prompted women-rights activists to demand changes. To increase public attention to the issue of domestic violence and its scope, women started stressing the serious and stigmatizing outcomes of the violence they experienced [18]. Barry describes this creation of a victim-image, which evokes pity and sympathy from outsiders, as "victimism" (18, p. 44). The price that battered women paid for the public regard they received consisted in their acceptance of a victim role and in the associations that this role carried, especially passivity, or—as Barry expressed it—being "the simple object of abuse without response whatever that response may be" (18, p. 46). This "victimization" led to an objectification and the negation of an active and evolving self [18, 19]. Positive traits, such as activity and strength, were insufficiently attributed to victims because of a focus on passive and stigmatizing effects of the experienced violence. This negative image became increasingly entrenched in the public perception, not least because of its use in the media, which depicted "passive victims" (16, p. 4) as sufferers unable to overcome their experiences [19]. Given the negative connotations that the label carries it is understandable that many women who were raped tend to avoid using the term in self-reference.

Other than the label "victim", the term "survivor" potentially carries attributes such as agency and initiative [18]. The label "survivor" thus emphasizes an orientation toward active resistance and recovery [16] as well as a rejection of ascribed passivity [19]. These connotations are often built upon in therapeutic work with women who were raped. Creating a "survivor"-identity is seen as an attempt to focus on a person's strengths and to support their capability to cope with their experience [20]. Women with a "survivor"-identity, as Mills [21] points out, focus on positive aspects and ways to change and carry on with their lives.

To date, few researchers have specifically addressed the two labels empirically. Skjelbaek [22] reports about qualitative interviews with women who had repeatedly experienced sexual violence during the war of Bosnia and Herzegovina between 1992 and 1995. Many of these women chose to regard themselves not only as "victims" but also as "survivors" of their experiences, as they withstood violence and found their way back into a normal life. Leisenring [17] interviewed women who experienced psychological or physical violence in their relationships. Some of these women rejected labeling themselves as "victims" because of the negative connotations of this term. Yet others reported explicitly referring to the "victim"-label in order to express the fact that they have suffered and deserve sympathy and redemption. In sum, about 75% of the interviewees explicitly identified at least partly with the image of the "victim". A quantitative study investigating the labels is reported by Park, Zlateva, and Blank [23], who interviewed cancer patients regarding their preferences for the use of various self-definitions. The patients indicated how much they identified with the terms "patient", "person who has had cancer", "victim", and "survivor". The results showed that 83% of the participants identified at least partly with the term "survivor", whereas only 18% partly identified with the term "victim". Furthermore, those with a "survivor"-identity more actively faced up to their diagnosis, reported higher well-being and less negative affect than those with a "victim"-identity.

In a paper on "victims" and "survivors" of crime, Boyle [24] reports that the "victim"-label was more strongly associated with mental illness, post-traumatic stress disorder, and feelings of shame, whereas the "survivor"-label went along with feelings of anger and a personal distancing from negative experiences. According to Thompson [13], women who were raped may vary the use of the labels or choose to use no label at all depending on the specific context, the reaction they intend to evoke, and the associations they want to stress. Thompson explicitly refers to the dilemma of conflicting consequences of the labels' use (what she calls the "victim-survivor-paradox"), where the aim of making perceivers take the rape seriously may conflict with the need to cope with its effects and overcome it (13, p. 329).

In a recent series of studies, Hockett, McGraw, and Saucier [25] explicitly examined characteristics associated with both the "victim"- and the "survivor"-label as well as potential effects on rape-related perceptions. In their studies, Hockett et al. illustrate that the "victim"-label is strongly associated with descriptions of stable personal attributes whereas the "survivor"-label refers to descriptions of outcomes and processes-, suggesting that women experiencing sexual violence are initially made "victims" but can subsequently become "survivors" when successfully coping with the experience. Regarding the attribution of responsibility and blame, Hockett and colleagues observed a gender difference, with male (vs. female) participants attributing more responsibility to the perpetrator if the woman was referred to as "survivor". Differences were less distinct regarding the valence of connotations ascribed to the labels. The terms significantly differed concerning the number of positive characteristics participants ascribed to them, with the "survivor"-label being associated with substantially more positive characteristics than the "victim"-label, but no differences emerged regarding the number of neutral or negative attributes assigned. Examining the labels’ effects on the perception of rape cases and their outcomes, Hockett et al. report specific effects on the attribution of blame but do not find evidence for effects on other measures of rape outcome perception. In her dissertation, Hockett [26] furthermore examined the labels' effects on the intention to support women who were raped, on an intergroup as well as an interpersonal level, but did not find evidence for the labels affecting these dependent variables.

### The present research

To summarize, theoretical discussions about the labels and their connotations as well as previous research findings suggest the existence of differences in meaning between the English terms "victim" and "survivor". Whether the use of these labels causes any clear-cut differences in the perception of rape cases, however, has not been unequivocally demonstrated yet. Also, the meaning of the terms in languages other than English has not been studied yet. Accordingly, our present work had the following aims: (1) To examine whether the proposed differences in meaning between the two labels can be shown using a classic instrument for the measurement of meaning, the s*emantic differential* [27]; (2) to explore further whether the two labels differentially affect perceptions of rape cases and of women who were raped, using short and relatively ambiguous vignettes in which the labels were either assigned by a third person or used by the woman to describe herself; (3) to examine the acceptance of the labels' use and potential factors influencing it (again in relation to either externally assigned or self-chosen labels); (4) to test whether the assumed differences are present across different languages, running all studies in parallel with English-speaking and German-speaking samples.

As judgments on the perception of rape cases and their outcomes are usually affected by participants’ characteristics and attitudes [7, 8], we decided to control for participants’ gender as well as their attitude toward feminism, using a short form of Morgan’s [28] Liberal Feminist Attitude and Ideology Scale (LFAIS). The questionnaire’s short form assesses pro-feminist attitudes and behavioral intentions. In our follow-up studies we additionally controlled for participants' acceptance of modern myths about sexual aggression (AMMSA) using an 11-item short form adapted from Gerger, Kley, Bohner, and Siebler [29]. Myths about sexual aggression, as defined by Gerger et al., are beliefs that deny, downplay, and justify sexual aggression perpetrated by men against women.

### Ethics statement

Procedures for all three studies reported below were approved by the Ethics Committee of the University of Bielefeld. To protect participants' anonymity, the ethics committee waived the need for written informed consent from the participants. After reading the study information on the Internet or on a lab computer, participants consented by clicking an "agree" button or by stating that they agree to continue, respectively, and their agreement was documented anonymously. Labeled datasets from all studies may be accessed without restriction from the PUB (Publications of Bielefeld University) database under https://pub.uni-bielefeld.de/data/2900097 (doi: 10.4119/unibi/2900097).

## Study 1

### Method

Our first study was conducted to examine the conscious connotations people associate with the English labels "victim" and "survivor" as well as with the equivalent German labels "Opfer" and "Überlebende". Parallel but separately accessible online surveys with identical content were conducted for an English- and a German-speaking sample.

#### Participants and design

Participants were recruited via posting the surveys’ links on social networks and Internet forums. The German language version was furthermore spread via handing the link out to students of the University of Bielefeld, Germany, while the English version was spread via personal contacts and colleagues to participants in the UK and the US, respectively. In sum, 86 participants (mean age = 22.98, *SD* = 5.56) completed the English version, and 93 participants (mean age = 25.46, *SD* = 6.57) completed the German version of the study. Within each language version, participants were randomly assigned to either a "victim" condition (*n* = 49 and 40, respectively, for the English and German versions) or a "survivor" condition (*n* = 37 and 53, respectively). Each sample consisted mainly of first-language speakers (English: 80.0%; German: 96.7%) and students (90.7% and 68.9%, respectively). The English sample mainly consisted of females (70.9%) while gender was equally distributed within the German sample. For those participants who were no first-language speakers, their mean age when learning the language was 7.59 years (*SD* = 3.74) within the English sample and 6.33 years (*SD* = 4.16) within the German sample.

#### Procedure and measures

The study was conducted as an online survey. Participants were informed that the study would deal with the topic of sexual violence and that participation was fully anonymous and voluntary. They also learned that they could withdraw their participation at any time simply by closing the browser window. Participants gave their informed consent by clicking an "agree" button. After reporting some demographic information, participants read a vignette in which a woman was raped by a stranger on her way back home from work. Depending on the experimental condition, the woman was referred to as either "victim" or "survivor" in the vignette's first sentence. The English version of the vignette read as follows:

"*Anna T*. *is a rape victim [survivor]*. *She works as an insurance saleswoman in Manchester*. *On a Tuesday evening she walks from her workplace to the parking area to drive home by car*. *As she searches for the keys in her handbag a stranger appears and asks her for the time*. *At the moment Anna looks at her watch*, *the man grabs her*, *drags her into nearby bushes and rapes her*. *Then he leaves the crime scene*."

After reading the vignette, participants were asked to rate the label presented in their condition using a s*emantic differential* [27] consisting of 15 bipolar pairs of adjectives that each defined a 7-point scale (e.g.: *hard*–*soft*; *bad*–*good*; *active*–*passive*). Participants were asked to mark, for each pair, the point that best represented their association with the label they had been presented with (*"Please indicate now how well the following adjectives describe the term ‘victim’/’survivor‴*). Based on the original studies on the s*emantic differential*, the adjectives used were designed to represent the three dimensions of evaluation, potency, and activity. Most pairs of adjectives were adapted from the original studies (e.g., *bad—good; active—passive*), but we also generated new pairs of adjectives that fitted our specific topic (e.g., *guilty—innocent; backward-looking—forward-looking*).

We additionally assessed participants' feminist attitudes using a short-form of Morgan’s [28] Liberal Feminist Attitude and Ideology Scale (LFAIS) consisting of 10 items [e.g., "*Although women can be good leaders*, *men make better leaders*"; response scale from 1, *totally disagree*, to 7, *totally agree*]. The scale’s German version was based on an adaptation by Bohner, Ahlborn, and Steiner [30]. Cronbach’s alpha was α = .79 for the English version and α = .73 for the German version.

### Results

#### Analysis plan

To assess the s*emantic differential'*s factorial structure, exploratory factor analyses were computed within each language sample, using maximum-likelihood extraction and varimax rotation, including the 15 pairs of adjectives. We later compared the labels' associations for each pair of adjectives as well as for composite scales based on the factor analyses. We additionally tested for interaction effects with participants' gender and feminist attitude using moderated regression analyses.

#### Factor analyses

Neither in the English nor in the German sample did we replicate the *semantic differential'*s three- factorial structure consisting of the factors evaluation, potency, and activity. Instead, in both samples we extracted a single main factor based on scree plots and the factors' eigenvalues. Within the English sample the scree plot suggested a single-factor solution with an eigenvalue of 6.91, explaining 46.1% of the dependent variable’s variance. Within the German sample the scree plot also suggested a single-factor solution with an eigenvalue of 6.67, explaining 44.5% of the variance. We thus averaged the 15 items into a one-dimensional scale representing a mix of *evaluation/potency/activity*. Cronbach's alpha for this scale was very good (English version: α = .89; German version: α = .90).

#### Semantic differential

**Fig 1:**
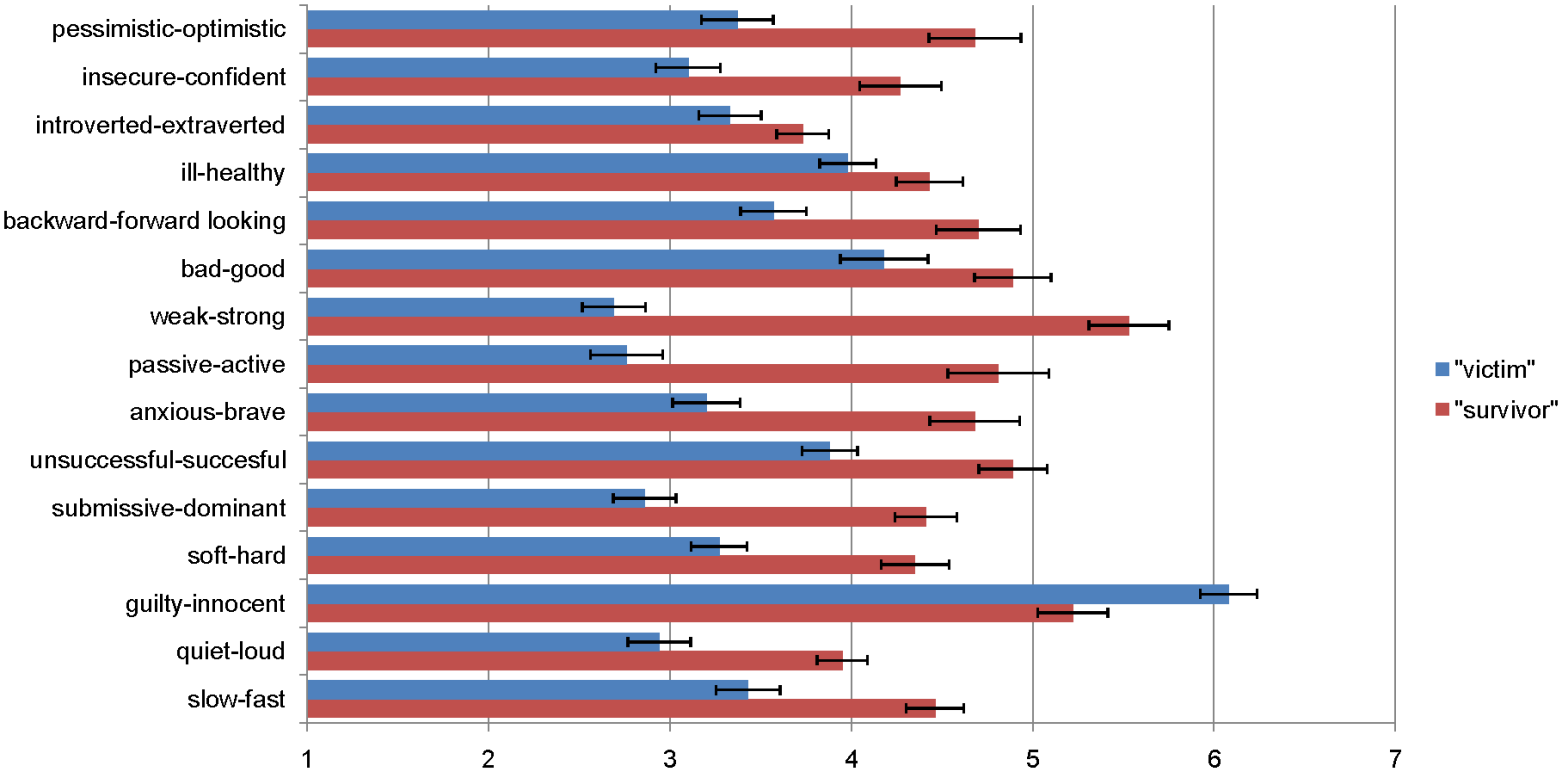
Means of semantic differential ratings by label condition (Study 1, English-language sample, *n* = 86). Error bars represent standard errors.

**Fig 2:**
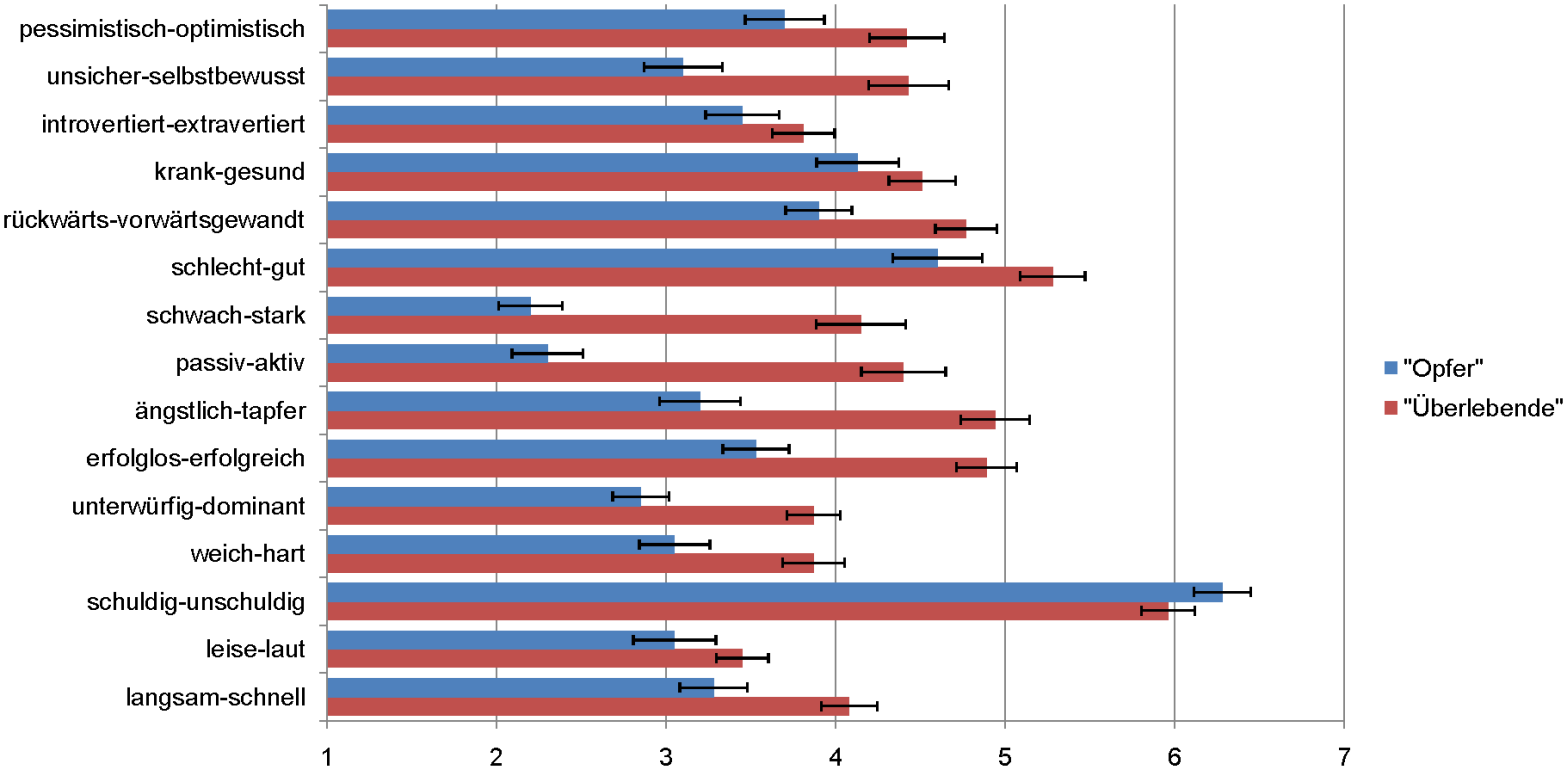
Means of semantic differential ratings by label condition (Study 1, German-language sample, *n* = 93). Error bars represent standard errors.

Results are displayed in Tables 1 and 2 as well as in Figs 1 and 2. *T*-Tests indicated that the label conditions strongly affected participants' conscious associations with the presented pairs of adjectives in both language samples. On the 15-item scale, the "survivor"-label was rated to be significantly more positive/strong/active than the "victim"-label for both the English version (*M* = 4.59, *SD* = 0.76 vs. *M* = 3.51, *SD* = 0.65), *t*(84) = 7.01, *p* = .001, *d* = 1.53, and the German version (*M* = 4.46, *SD* = 0.88 vs. *M* = 3.51, *SD* = 0.81), *t*(91) = 5.31, *p* = .001, *d* = 1.11. At the item level, as shown in Table 1, significant differences were found for 13 out of 15 items in the English version. Directionally, all ratings were more positive/strong/active for "survivor" than "victim", with the single exception that "victim" was associated more strongly with "innocent" (*p* = .001). The item results for the German version were highly similar (see Table 2): Significant differences were found for 11 out of 15 items. Directionally, all ratings were more positive/strong/active for "survivor" than "victim", with the single exception that "victim" was nonsignificantly associated more strongly with "innocent". Moderated regression analyses showed that most of these effects were independent of participants' gender or feminist attitude, with just two exceptions in the English sample: The difference for "guilty-innocent" was significant only for female participants (*M* = 6.32 vs. 5.11; *p* < .001) but not for male participants (*M* = 5.53 vs. 5.50; p > .94), *t*(80) = 2.54, *p* = .013 for the interaction. Furthermore, the difference for submissive-dominant was more pronounced at higher levels of feminist attitude (1 *SD* above the mean: *M* = 2.73 vs. 4.89, *p* < .001) vs. lower levels of feminist attitude (1 *SD* below the mean: *M* = 3.07 vs. 4.15, *p* < .01), *t*(80) = 2.23, *p* = .028 for the interaction.

**Table 1:** Means and standard deviations (in parentheses) of semantic differential ratings by label condition, t-Test results and effect size estimates (Study 1, English-language sample).

	victim (*n* = 49)	survivor (*n* = 37)	*t*-test for difference (*df* = 84)	Cohen’s *d*
pessimistic—optimistic	3.37 (1.40)	4.68 (1.55)	4.11***	0.90
insecure—confident	3.10 (1.25)	4.27 (1.37)	4.13***	0.91
introverted—extraverted	3.33 (1.20)	3.73 (0.87)	1.73	0.38
ill—healthy	3.98 (1.09)	4.43 (1.12)	1.88	0.41
backward-looking—forward-looking	3.57 (1.28)	4.70 (1.41)	3.89***	0.86
bad—good	4.18 (1.69)	4.89 (1.29)	2.12*	0.47
weak—strong	2.69 (1.23)	5.53 (1.34)	9.56***	2.25
passive—active	2.76 (1.39)	4.81 (1.70)	6.00***	1.36
anxious—brave	3.20 (1.31)	4.68 (1.51)	4.84***	1.20
unsuccessful—successful	3.88 (1.07)	4.89 (1.15)	4.17***	0.92
submissive—dominant	2.86 (1.21)	4.41 (1.04)	6.24***	1.38
soft—hard	3.27 (1.08)	4.35 (1.14)	4.53***	0.99
guilty—innocent	6.08 (1.10)	5.22 (1.18)	3.47***	0.77
quiet—loud	2.94 (1.22)	3.95 (0.85)	4.53***	0.95
slow—fast	3.43 (0.82)	4.46 (0.96)	5.73***	1.18

*Note*. 7-point scales.

*p < .05,

**p < .01,

***p < .001

**Table 2:** Means and standard deviations (in parentheses) of semantic differential ratings by label condition, t-Test results and effect size estimates (Study 1, German-language sample).

	Opfer (*n* = 40)	Überlebende (*n* = 53)	*t*-test for difference (*df* = 91)	Cohen’s *d*
pessimistisch—optimistisch	3.70 (1.47)	4.42 (1.60)	2.20*	0.47
unsicher—selbstbewusst	3.10 (1.46)	4.43 (1.72)	3.95***	0.83
introvertiert—extravertiert	3.45 (1.38)	3.81 (1.33)	1.28	0.27
krank—gesund	4.13 (1.54)	4.51 (1.44)	1.24	0.26
rückwärtsgewandt—vorwärtsgewandt	3.90 (1.24)	4.77 (1.33)	3.24**	0.68
schlecht—gut	4.60 (1.66)	5.28 (1.39)	2.16*	0.45
schwach—stark	2.20 (1.18)	4.15 (1.93)	6.03***	1.19
passiv—aktiv	2.30 (1.32)	4.40 (1.81)	6.45***	1.31
ängstlich—tapfer	3.20 (1.51)	4.94 (1.49)	5.57***	1.17
erfolglos—erfolgreich	3.53 (1.24)	4.89 (1.30)	5.11***	1.08
unterwürfig—dominant	2.85 (1.05)	3.87 (1.14)	4.40***	0.94
weich—hart	3.05 (1.32)	3.87 (1.33)	2.95**	0.63
schuldig—unschuldig	6.28 (1.06)	5.96 (1.14)	-1.34	-0.29
leise—laut	3.05 (1.54)	3.45 (1.12)	1.46	0.31
langsam—schnell	3.28 (1.26)	4.08 (1.19)	3.13**	0.66

*Note*. 7-point scales.

*p < .05,

**p < .01,

***p < .001

### Discussion

The results of Study 1 indicate that observers perceive the term "survivor" (and its German equivalent) as indicating more positivity, strength, and activity than the term "victim" (and its German equivalent). The fact that the three-factorial structure of the *semantic differential* could not be replicated suggests that the three dimensions may be closely related in this content area, such that a "survivor" is inherently seen as better *and* stronger *and* more active compared to a "victim". These results complement and extend previous findings by Hockett et al. [25], who had used a different methodology but also found ascriptions of *strength* to distinguish "survivors" from "victims". In their study, Hockett and colleagues asked participants to openly report five characteristics they associate with either the term "rape victim" or the term "rape survivor". Whereas the "victim"-label was associated with characteristics such as *afraid* and *distrusting*, but also with positive terms such as *attractive*, the "survivor"-label was associated more with terms such as *strong* and *angry*, but also with negative terms such as *afraid*.

Our results also show that other positive attributes such as activity and optimism are associated with the labels not only by rape survivors themselves but also shared by outside observers reading about a case of rape. Conversely, the term "victim" was rated to be less positive, less strong, and more passive overall, illustrating that unfavorable connotations are associated with this label also for outside observers. Solely the English item "*guilty—innocent*" showed a significantly more positive association for "victim" than for "survivor". This may be explained by the common everyday expression "innocent victim", which may have triggered this associative connection, and replicates the results of previous studies [13].

Of interest, our results were highly comparable for both language versions. This suggests that similar meanings are attached to the two labels in the English and German languages. The clear distinctness of the labels' connotations is illustrated by the effect sizes examined for the comparison of means, which, according to Cohen [31], can be regarded as large in both language versions.

## Study 2

Our second study aimed at an exploration of the labels' potential effects on the perception of rape cases. We decided to implement a different methodology compared to the studies reported by Hockett and colleagues [25], as we felt that the emergence of distinct label effects on the perception of rape-related outcomes may partially depend on the methods applied. The rape vignettes used by Hockett et al. were rather detailed and unambiguous regarding the attribution of responsibility, thus potentially limiting the labels' influence on participants' judgments. By contrast, we decided to present a rape vignette that was less detailed and also less obvious, describing a more ambiguous course of events. Furthermore, we suspected that the label manipulation of Hockett and colleagues may have been too weak to affect participants' judgments, which is supported by the fact that only 64.6% of participants correctly remembered the presented label in their first preliminary study. We therefore tried to increase the labels' salience, following studies on leading questions by Loftus and Palmer [32]. In their original studies, Loftus and Palmer demonstrated how the variation of a single word within an item could significantly change participants' reconstruction of a previously witnessed event. For instance, when asked how fast two cars previously seen in a film were when they "smashed into" each other, participants estimated higher speeds than when asked how fast the cars were when they "hit" each other. We adapted this method, presenting the labels "victim" or "survivor" within items asking about a rape that participants had previously read about. Using this method ensures that the labels are presented in a subtle way and that they are permanently salient while participants make their judgments.

### Method

#### Participants

The English version again was conducted as an online survey, and the link was spread via social networks, Internet forums as well as personal contacts and colleagues in the UK and the US. The German version was conducted as a laboratory study; participants were recruited at the University of Bielefeld and received sweets for their participation. The content of the studies was identical in both language versions. In sum, 37 participants (mean age = 24.84, *SD* = 9.08) completed the English version and 58 participants (mean age = 23.22, *SD* = 2.64) completed the German version. Participants were randomly assigned either to the "victim" condition (*n* = 16 and 29, respectively, for the English and German versions) or to the "survivor" condition (*n* = 21 and 29, respectively). Both samples consisted mainly of students (77.8% for the English-language sample; 98.3% for the German-language sample), and the gender distribution was roughly equal in both samples (54.1% and 50% males, respectively).

#### Procedure and measures

As in our first study, participants gave their informed consent after learning that the study was fully anonymous and participation was voluntary. Participants were informed that the study's topic was sexual violence and that they were free to skip the part explicitly dealing with the description of a rape. The data of seven participants skipping this part were excluded from analyses, leaving the final numbers of participants reported above. If participants decided to complete the entire study, they read a vignette describing a case of acquaintance rape in which a woman was raped by a coworker after a dinner with sexual innuendos on both sides. The English version of the vignette read as follows:

"*On a Tuesday evening*, *at the end of the day's work*, *a woman prepares for her way home when she is approached by a colleague*. *He invites her to go out for dinner after work*. *She does not know him well*, *but he appears to be likable and so she agrees*. *They leave their workplace together and proceed to a nearby restaurant*. *After a nice dinner and a good conversation he suggests to go for a walk*. *In the course of the evening the man repeatedly makes innuendos and invites the woman to accompany him to his place*. *She feels flattered by his approaches*, *returns his innuendos and*, *being in the mood*, *she kisses him*. *They keep walking for a while and then decide to end the evening*. *The man asks her again to come with him to his flat*. *She agrees to have a coffee*. *On their way the man repeatedly drops sexual hints*, *which she returns*, *and again she kisses him*. *Having arrived at his flat they have a coffee and chat; then she decides to go home*. *The man tells her to stay overnight again and again*, *but she declines*, *gives him a kiss and heads for the door*. *Before she can leave the flat*, *the man seizes her*, *drags her into his bedroom and rapes her*. *When he finally lets up on her*, *she is able to grab her clothes and leave the flat*."

After reading the vignette, participants were asked to answer several items regarding their perception of the rape and the persons involved. The wording of these items varied depending on the experimental condition: The woman was consistently referred to as "victim" or "survivor" in the English versions, and as "Opfer" or "Überlebende" in the German versions.

Items assessing the perception of the rape measured the perceived responsibility of the woman ("*Does the victim [survivor] herself carry a certain responsibility for what happened*?"; "*How likely do you think it is that the victim [survivor] could have prevented the rape from happening*?"; "*How appropriate do you think it is that the victim [survivor] presses charges against the perpetrator*?" (reverse-coded)), the perceived outcome severity ("*How likely do you think it is that the victim [survivor] will need social support to recover from the rape*?"; "*How likely do you think it is that the victim [survivor] will suffer from psychological problems as a result of the rape*?"; "*How likely do you think it is that the victim [survivor] suffered physical injuries as a result of the rape*?") and the appropriateness of support ("*How likely do you think it is that the victim [survivor] fully recovers from the event*?" (reverse-coded); "*How appropriate do you think it is that the victim [survivor] sought psychological help after the rape*?"; "*How appropriate do you think it is that the victim [survivor] claims an injury award*?"). All items were answered on a 9-point scale, from 1, *very unlikely* or *very inappropriate*, to 9, *very likely* or *very appropriate*.

For exploratory purposes, participants were furthermore asked to judge the physical appearance of both the woman and the perpetrator, rating their age, body height, attractiveness, and stature (each on category scales from 1, *lowest*, to 9, *highest*) as well as imagining their clothing style (by selecting one of five options: *messy*, *casual*, *sporty*, *elegant*, *sexy*). Again, the specific label was integrated into the items pertaining to the woman.

Finally, participants were asked if the specific label they had received was appropriate to refer to the woman ("*Do you think the use of the label 'victim' ['survivor'] is appropriate in this situation*?"), response options were "*yes*" and "*no*". Participants who judged the label to be inappropriate were additionally asked to state an alternative label that they would regard as more appropriate.

Again we assessed participants' feminist attitudes using the 10-item LFAIS [28]; Cronbach’s alpha for this scale was .87 for the English-language sample and .65 for the German-language sample. Additionally, we assessed participants' acceptance of modern myths about sexual aggression (AMMSA) using an 11-item short form adapted from Gerger and colleagues [29] (e.g. "*Many women tend to misinterpret a well-meant gesture as a 'sexual assault‴*). Myths about sexual aggression, as defined by Gerger et al., are beliefs that deny, downplay, and justify sexual aggression perpetrated by men against women. Items were answered on a 7-point scale from 1, *totally disagree*, to 7, *totally agree*. Cronbach's alpha was .93 for the English-language sample and .85 for the German-language sample.

### Results

#### Analysis plan

The 3-item scales assessing *woman's responsibility*, *outcome severity*, and *appropriateness of support*, respectively, had internal consistencies ranging from α = .56 (*appropriateness of support*, German sample) to α = .85 (*perceived outcome severity*, English sample). We conducted 2-way ANCOVAs to test for effects of label condition, gender, and their interaction, with participants’ feminist attitude and AMMSA as covariates; all means reported will be adjusted for effects of the covariates. We note that cell sizes in our design were unequal, and some were small. However, statistical research has shown that F-tests in ANCOVA are robust; specifically, Type I error rate is well controlled even for small, unequal sample sizes and with non-normal distributions of residuals, as long as the homogeneity of variance assumption is met [33]. In our Studies 2 and 3, homogeneity of variances could be assumed for all analyses reported (Levene tests, all *p* > .05), and normality of residuals was also generally given (Kolmogorov-Smirnov test, all *p* > .05), except in two cases. For Study 2 residuals were not normally distributed for the scale of *perceived outcome severity* within the German-language sample, *D*(58) = 0.16, *p* = .001. Condition effects on the categorical judgments of clothing style as well as on the perceived appropriateness of the labels were tested via chi-square analyses.

#### Perception of the rape and personal attributes

Within the English-language sample, the ANCOVA revealed a significant interaction effect of label and gender on perceived outcome severity, *F*(1, 31) = 10.78, *p* = .003, η^2^ = .26. Female participants perceived the outcomes of the rape as more severe when the woman was referred to as a "survivor" (*M* = 7.95) rather than a "victim" (*M* = 6.50), *F*(1, 31) = 4.36, *p* = .045, η^2^ = .12. Male participants, by contrast, perceived the outcomes of the rape as *less* severe when the woman was referred to as a "survivor" (*M* = 6.16) rather than a "victim" (*M* = 7.80), *F*(1, 31) = 6.48, *p* = .016, η^2^ = .17. The covariates included had some significant effects on the outcome variables: While participants' AMMSA was correlated with the woman's perceived responsibility, *r* = .59, *p* < .001, participants' feminist attitude was correlated with perceived outcome severity, *r* = .53, *p* = .001.

Within the German-language sample, the ANCOVA revealed significant effects on perceived appropriateness of support: Main effects of label condition, *F*(1, 52) = 3.98, *p* = .051, and gender, *F*(1, 52) = 5.58, *p* = .022, were qualified by a significant interaction effect of label and gender, *F*(1, 52) = 7.25, *p* = .010, η^2^ = .12. Female participants perceived support for the woman as equally appropriate when she was referred to as a "survivor" (*M* = 7.75) as when she was referred to as a "victim" (*M* = 7.90), *F* < 1. Male participants, by contrast, perceived support for the woman as more appropriate when she was referred to as a "survivor" (*M* = 7.79) than when she was referred to as a "victim" (*M* = 6.46), *F*(1, 52) = 10.99, *p* = .002, η^2^ = .17. Furthermore, participants' AMMSA was significantly related to the woman’s perceived responsibility, *r* = .45, *p* < .001.

In exploratory analyses, we did not find any significant main or interaction effects involving label condition on participants' judgments of the perpetrator's appearance or clothing style, all p > .15. Also, there were no effects involving label condition on the woman's perceived height, stature, or age, all *p* > .15. For the woman's perceived attractiveness, the ANCOVA on the English-language sample revealed a main effect of gender, *F*(1, 31) = 9.66, *p* = .004, which was qualified by a significant interaction of label by gender, *F*(1,31) = 4.87, p = .035, η^2^ = .14. Men judged the raped woman to be more attractive (*M* = 7.00) than women did (*M* = 5.66), and this was especially pronounced in the "victim" condition (*M* = 7.39 vs. 5.14), *F*(1,31) = 12.24, p = .001, but less so in the "survivor" condition (*M* = 6.62 vs. 6.18), *F* < 1. The covariate LFAIS was positively correlated with perceived attractiveness of the woman, *r* = .39, *p* = .018.

An initial inspection of choices regarding the woman's clothing (from the categories *casual*, *elegant*, *sexy*, *sporty*, and *messy*) showed that the two categories most often chosen overall were "casual" and "elegant", together accounting for about 77% of responses. Interestingly, most participants in the "victim" condition imagined the woman to be *casually* dressed (50% and 48% in the English and German samples, respectively), whereas most participants in the "survivor" condition imagined the woman to be *elegantly* dressed (57% and 48%). Although this pattern was not significant in either language sample on its own, when we combined the two samples for a focused table analysis, we found a significant result ("victim": casual 48.9%, elegant 28.9%, other 22.2%; "survivor": casual 22.0%, elegant 54.0%, other 24.0%), χ^2^ (3; *N* = 95) = 9.07, *p* = .028, N = .31.

#### Appropriateness of the labels

The dichotomous ratings of the labels’ appropriateness differed significantly and strongly in each language sample. Within the English sample, 14 out of 16 participants (87.5%) judged the "victim"-label to be appropriate, whereas only 8 out of 21 (38.1%) participants judged the "survivor"-label to be appropriate, χ^2^ (1; *N* = 37) = 9.20, *p* = .001, N = .50. Within the "survivor" condition, most of the participants rejecting this label recommended using the term "victim" instead (76.9%). Likewise, within the German sample, 28 out of 29 participants (96.6%) judged the "Opfer"-label to be appropriate, whereas only 8 out of 29 participants (27.6%) judged the "Überlebende"-label to be appropriate, χ^2^ (1; *N* = 58) = 29.29, *p* = .001, N = .71. Within the "Überlebende" condition, most of the participants rejecting this label recommended using the term "Opfer" instead (71.4%).

### Discussion

Our second study shed some light on the labels' potential effects on the perception of a rape case and the persons involved. Contrary to our expectations based on the connotations examined in Study 1, the labels showed only few main effects on participants' perception of the rape. Instead, we found some interactions of label by gender, which reflected that men tended to be more strongly affected by the labels than women overall, and sometimes effects were opposite for men than for women: The English term "survivor" increased the perceived severity of the rape and its outcomes when rated by female participants, whereas male participants perceived the "victim's" experience to be more severe. Furthermore, those effects of the label condition that we did observe were not parallel in the two language samples. These relatively weak and mixed findings resemble those of Hockett et al. [25]: Despite our use of a more ambiguous vignette and a more salient way of presenting the labels, we did not find clear-cut effects of the labels on participants' judgments, although the significantly differing connotations we examined in Study 1 would have suggested so.

A potential explanation for this lack of effects may lie in participants' ratings of the labels' appropriateness. In both the English and the German sample, the "survivor" label was rated as clearly inappropriate to refer to the woman in the given context. This rejection of the "survivor" label may be explained by two factors that we had mentioned in the Introduction: implications of severity, and time perspective. Firstly, the term "survivor" (as opposed to "victim") may imply that an experience has been (at least potentially) life-threatening. Participants may not have perceived this kind of lethal threat in the acquaintance-rape vignette they read and may thus have regarded the "survivor" label as inappropriate and somewhat dramatizing. Secondly, as the dependent variables focused on the rape's short-term outcomes, participants may have perceived it more appropriate to use "victim" as a default label rather than to use "survivor" which may have been more appropriate to refer to the woman's long-term coping with her experience or to an assumed identity. In any case, the strong rejection of the label's use may have dampened any effects it may have had on the perception of the rape. Additionally, the partially low Cronbach’s alphas of the LFAIS (German sample) as well as the 3-item scales we assessed may have contributed to the inconsistent findings.

At the same time, the strong correlations of AMMSA and LFAIS with judgments of the rape case, which replicate previous research [8, 9, 34], indicate that the vignettes used were suitable in principle to study differences in subjective perceptions. Together, these results suggested that it might be possible to find an influence of the labels under study on perceptions of a rape case, given that the labels are presented in such a way that they are both regarded as appropriate. This assumption was additionally supported by the suggestive pattern we found in exploratory analyses of the woman's clothing style that participants imagined. Here, the labels appeared to influence not only the perception of the rape but also the image of the woman who was raped. Based on these considerations we set out to further investigate the labels' effects in a third study using a modified paradigm to overcome issues we experienced in Study 2. Specifically, we aimed at leaving the severity of the rape more ambiguous and placing the emphasis on its long-term rather than short-term outcomes.

## Study 3

In our third study we decided to switch to a different paradigm to focus on the aftermath of rape and a woman’s coping with the negative experience. We therefore presented the "survivor" and "victim" labels each as a self-reference chosen by the woman herself (rather than as being chosen by someone else). Our assumption was that both labels would be regarded as more appropriate if presented from a first-person perspective, and that potential effects of the labels on participants' perception of the rape would therefore become more likely to occur. This aspect of third- vs. first-person ascription of the labels has not been investigated explicitly in previous research. Furthermore, we assumed that the labels' influences would be more distinct when we focus on the woman's long-term recovery and her processing of the negative experience, instead of focusing on the rape's more immediate outcomes. Finally, to further increase the perception of the labels as appropriate, we decided to reduce information on the actual rape scenario, avoiding the issues we faced in Study 2 and leaving more room for interpretation of the rape and its circumstances. Therefore, we presented the woman's statement as having been made a few months after the rape experience and included no details on the rape itself.

### Method

Again parallel but separately accessible online surveys with identical content were conducted for an English- and a German-speaking sample.

#### Participants and design

As for Study 1, participants were recruited via social networks, Internet forums as well as handing the link to the German version out to students of the University of Bielefeld and spreading the link to the English version via personal contacts and colleagues in the UK and the US. In total, 190 participants took part in the third study; of these, 50 completed the English version (mean age = 30.26, *SD* = 10.06) and 140 completed the German version (mean age = 25.87, *SD* = 8.00). Participants were randomly assigned either to the "victim" condition (*n* = 33 and 68, respectively, for the English and German versions) or to the "survivor" condition (*n* = 17 and 72, respectively). Again, most participants were students (70.0% within the English- and 80.7% within the German-speaking sample). While the English sample mainly consisted of male participants (68.0%) the German sample mainly consisted of females (73.6%).

#### Procedure and measures

Both language versions were conducted as parallel online surveys. After learning that participation was anonymous and voluntary, and that the study dealt with the topic of sexual aggression, participants gave their informed consent electronically. Then they were shown a vignette in which a woman who had allegedly been raped some time ago talks about the importance of choosing a role for her future life. Therefore the labels were not presented as applied by a third person, but as a self-chosen identity. Depending on the condition, the vignette ended with a self-reference as either "victim" or "survivor". The English version read:

"*Especially the time after the event has not been easy*. *I had to tell what happened over and over again*. *Such an experience can leave its marks*. *You ask yourself a lot of questions*. *How could it happen*? *Why me*? *For me and my future it has been essential to make a decision and choose which role to take in all of this*. *Do I want to be weak or strong*? *Active or passive*? *Do I let it get me down or take my life into my own hands*? *Do I look into the future optimistically or live in the past*? *To me it is clear*: *He is the perpetrator*. *And I am the victim [survivor]*!"

Items assessing the perception of the rape measured the woman's psychological stability ("*How optimistic do you think the woman is*?"; "*How self-confident do you think the woman is*?"; "*How psychologically stable do you think the woman is*?"; "*How likely do you think it is that the woman tried to commit suicide after the event*?" (reverse-coded); "*How much do you think the woman has been traumatized in the weeks after the event*?" (reverse-coded), "*How likely do you think it is that the woman is active in groups fighting sexual violence*?"; "*How likely do you think it is that the woman sought psychological help after the event*?"), the probability of active resistance ("*How likely do you think it is that the woman actively resisted the perpetrator*?"; "*How much do you think the woman physically resisted the perpetrator*?") and the perceived outcome severity ("*How likely do you think it is that the woman suffered physical injuries as a result of the rape*?"; "*How likely do you think it is that the woman fully recovers from the event*?" (reverse-coded); "*How likely do you think it is that the woman's life was in danger during the event*?"; "*How likely do you think it is that the woman tried to suppress what happened*?"). All items were answered on 7-point scales, from 1, *very unlikely* or *not at all*, to 7, *very likely* or *very much*.

Finally participants rated, on a 7-point scale, the appropriateness of the specific label as a self-reference for the woman in the given situation ("*How appropriate do you think it is that the woman called herself a victim [survivor]*?"). If the label was rated as inappropriate, participants were asked to name an alternative label that they would regard as more appropriate.

Again we assessed participants' feminist attitudes, using the 10-item LFAIS-scale [28], as well as their AMMSA, using an 11-item short form based on Gerger et al. [29]. Cronbach's alpha for LFAIS was α = .74 for the English and α = .64 for the German sample. Cronbach's alpha for AMMSA was α = .90 for both the English and the German sample.

### Results

#### Analysis plan

The scales assessing the woman's *psychological stability*, *probability of active resistance*, and *outcome severity*, respectively, had internal consistencies ranging from α = .48 to α = .91. We conducted 2-way ANCOVAs to test for effects of label condition, gender, and their interaction, with feminist attitude and AMMSA as covariates. As mentioned before, homogeneity of variances could be assumed for all analyses reported. For Study 3 residuals were not normally distributed for the scale of *probability of active resistance* within the German-language sample, *D*(140) = 0.083, *p* = .021.

#### Perception of the rape

Within the English-language sample, we found a significant main effect of label condition on the woman's perceived psychological stability, *F*(1, 44) = 6.01, *p* = .018, η^2^ = .12. When the woman referred to herself as "victim", participants perceived her to be significantly less psychologically stable (*M* = 4.10) that when she referred to herself as "survivor" (*M* = 4.75). Furthermore, there was a trend toward an interaction of label and participants' gender regarding the perceived outcome severity, *F*(1, 44) = 3.45, *p* = .070, η^2^ = .07. When the woman referred to herself as "victim", female participants perceived the rape's outcomes to be more severe (*M* = 5.10) than did male participants (*M* = 4.28), *F*(1, 44) = 4.99, *p* = .031, η^2^ = .10. No such gender difference emerged within the "survivor" condition (*M* = 4.31 vs. 4.63), *F* < 1. The simple main effects of label were not significant for either gender, both *p* > .12. The perceived probability of active resistance showed only an effect of the covariate AMMSA (*r* = .33, p = .021) but no effects involving label condition or gender, all *F* < 1.

Within the German-language sample, we found a trend toward an interaction effect of label condition and gender on the probability of active resistance, *F*(1, 134) = 3.44, *p* = .066. When the woman was referred to as a "Opfer", female participants perceived active resistance by the woman to be more probable (*M* = 4.81) than did male participants (*M* = 3.94), *F*(1, 134) = 5.48, *p* = .021, η^2^ = .04. No such gender difference emerged within the "Überlebende" condition (*M* = 4.44 vs. 4.50), *F* < 1. The simple main effects of label were not significant for either gender, both *p* > .15. No effects involving label condition or gender were found for either perceived severity or the woman's psychological stability, all *p* > .27.

#### Appropriateness of the labels

In this study, the perceived appropriateness of both labels, now being used by the woman as a self-reference, was generally high (above 5 on a 1–7 scale). Nonetheless, we still observed trends toward greater perceived appropriateness of using "victim" rather than "survivor". In the English-speaking sample, the ANCOVA showed that this trend was nonsignificant ("victim": *M* = 6.04; "survivor": *M* = 5.36), *F*(1, 44) = 2.72, *p* = .106, η^2^ = .06.

In the German-speaking sample, the ANCOVA showed a main effect of label condition ("Opfer": *M* = 6.16; "Überlebende": *M* = 5.08), *F*(1, 134) = 13.41, *p* < .001, η^2^ = .09, which was qualified by an interaction of label condition and gender, *F*(1, 134) = 4.18, *p* = .043, η^2^ = .03: Whereas male participants judged "Opfer" to be more appropriate (M = 6.70) than "Überlebende" (*M* = 5.02), *F*(1, 134) = 11.03, *p* = .001, η^2^ = .08, there was no significant difference for female participants (*M* = 5.62 vs. 5.14), *F*(1, 134) = 2.52, *p* = .11, η^2^ = .02.

### Discussion

Our third study was designed to explore the labels' effects when used as a self-reference by a woman who had experienced sexual violence and now describes her coping with the experience. Within the English-language sample, our results revealed positive effects of the label "survivor", which increased the woman's perceived long-term psychological stability. In contrast to the results of Study 2, male participants perceived the "survivor’s" experience to be more severe, whereas female participants tended to perceive the "victim's" experience as more severe. Within the German-language sample, men tended to infer that the "survivor" showed more resistance, whereas women tended to infer that the "victim" showed more resistance. As discussed within the Introduction section, results may be explained by the differing meanings the term "survivor" can carry depending on whether it is perceived as stressing the physical invasiveness of an assault or the woman’s subsequent coping with the experience. While the paradigm of Study 3 was meant to highlight the latter, male participants within the English-language subsample may have used the term to estimate the cruelty of the rape when asked to rate the perceived outcome severity, as the vignette itself did not provide explicit information on the circumstances of the assault and its physical impact. Furthermore, the use of both labels as a self-reference was generally regarded as highly appropriate by our participants. This stands in contrast to Studies 1 and 2, where the term "survivor", when used by third parties, was seen as clearly inappropriate. In Study 3, within the English-language sample the term "survivor" was regarded as an almost equally appropriate self-reference as was the term "victim" in the given context. We should note, however, that the sample size for this comparison was small. Within the German-language sample, male participants judged the self-referential use of the label "Überlebende" to be less appropriate than the use of "Opfer".

## General discussion

Our research confirms that the positive connotations ascribed to the term "survivor" in theoretical discussions [16, 17] and by women who were raped are shared by outside observers when asked directly (Study 1). Overall, "survivor" was associated with positive valence, activity, strength, and optimism, whereas "victim" was associated more with negative valence, passivity, weakness, and helplessness. Differences in the perception of the terms were large, and the pattern of connotations replicated across different languages, as there were parallel effects for the equivalent terms in German, "Überlebende" and "Opfer".

The labels' effects on the actual perception of sexual violence and its outcomes turned out to be less pronounced (Studies 2 and 3). When presenting the labels in the form of leading questions (Study 2), they had little effect on participants' perception of the rape and its short-term outcomes, which, as our assessment of the labels' appropriateness underlines, may be explained by the low acceptance of the term "survivor" (and even lower acceptance of "Überlebende") when used by researchers in the context of an acquaintance rape scenario. Nonetheless, some effects of the labels were observed, but these were often inconsistent across participant genders and language samples. An exception is the strikingly similar effect of the labels on the clothing style ascribed to the woman: The "survivor" was generally seen as more "elegant", the "victim" as more "casual". These ascriptions may indicate perceived differences in status and power that mirror the semantic-differential findings of Study 1.

With the aim of increasing the acceptability of the "survivor" label in Study 3, we changed the mode of presentation of the labels from being assigned by a third person to being self-chosen identities in the aftermath of the rape, and thus put the emphasis on long-term rather than short-term outcomes of the experience; we also did not include any detail on the rape in the vignettes presented in Study 3, so as to leave room for inferences about the severity and potentially life-threatening nature of the woman's experience. This indeed led to greater acceptance of the label, and now both labels were regarded as highly appropriate. Nonetheless, the German term "Überlebende" was still regarded as less appropriate in this context than the term "Opfer" by male participants. Although effects of the self-referential labels on perceptions of the rape were overall weak and somewhat mixed, one clear result in line with the perceived meanings of Study 1 was that English-speaking participants perceived the "survivor" as more psychologically stable than the "victim" when focusing on long-term outcomes.

The differences between language samples that we observed may be explained by the differing conventionality of the terms' use in the context of sexual aggression. While in the English language both the term "victim" and the term "survivor" may be regarded as relatively established labels to refer to women experiencing sexual violence, the German term "Überlebende" appears to be less common and not yet established in discourse about sexual aggression. Internet searches we undertook for the relative frequency of the terms confirm this assumption. The additional effects we report for participants' feminist attitude and acceptance of sexual aggression myths in Studies 2 and 3 replicate previous research [2, 35, 5, 34, 7].

### Limitations and future directions

Although our results appear promising, some limitations need to be considered. Some of the sample sizes were small, especially for the English language versions of Study 2 and 3, so results should be interpreted cautiously and comparisons between the language versions should be drawn with the differing sample sizes in mind. Despite the robustness of the statistical analyses we employed [33], larger sample sizes would have been desirable in order to draw more reliable conclusions from hypothesis tests and should be attained in future studies. Furthermore, our samples mainly consisted of students, which may limit the results' generalizability. As Rogers and Davies [36] have shown, students tend to have a strong pro-victim perception when rating rape cases, perceiving victims as highly believable and perpetrators as highly responsible, thus variances may have been constrained by the samples' selectivity. Future studies examining the labels should not only ascertain the replicability of our results in larger and more diverse samples, but should also further investigate the context-dependence of the labels’ perception and effects and factors influencing the perceived appropriateness of their use. Nonetheless we regard our results as promising and as a sufficient foundation to draw first conclusions and derive ideas for further research.

We assume that when explicitly focusing on a rape's objective and immediate physical outcomes, the terms "survivor" and "Überlebende" may increase the perceived severity of the rape because they stress its potentially life-threatening impact. If this implied impact appears contradictory to the scenario presented, as may have been the case for the acquaintance rape scenario in Study 2, the labels’ may be regarded as inappropriate. By contrast, when focusing on a rape's subjective and long-term outcomes, especially a woman's coping with the experience, the labels can decrease the perceived severity and increase the perceived stability as they stress the person's strength, well-being, and successful coping with the rape. Future research should further investigate the factors influencing whether the term is perceived as either emphasizing a woman’s coping process or the assault’s severity.

Another aspect we consider highly interesting for future research is examining the labels' effects in the contexts of legal decision making and the endorsement of social and medical support. According to Krahé [37], a prototypical and believable case of rape includes serious physical and psychological consequences, and "legitimate" victims are perceived as helpless and passive. If this stereotypical image is challenged, the support granted to the woman and the sentence demanded for the perpetrator tend to decrease [38]. The "survivor" (or "Überlebende") label, if perceived as emphasizing a positive coping process, may call these stereotypical images into question and may thus undermine a fair treatment of women who were raped.

Beyond exploring conscious connotations and effects of the labels, future research should also address implicit associations evoked by those labels, using methods such as the implicit association test [39]–for an application to rape perceptions, see [40]. Also, future studies should investigate not only outsiders' perception of the labels but also focus on the way women who were raped interpret and use the labels themselves, as well as on factors determining the choice of a specific self-reference in a specific context. We assume that the somewhat dichotomous view of self-labeling as "victim" *or* "survivor" does not do justice to the complex question of defining and evolving a self-concept after experiencing sexual violence and that for women concerned there is a lot more to the topic than a general choice of either one label or the other. Finally, connotations and effects of the labels should be explored in other languages as well, as our data have highlighted both similarities and differences between the English and the German terms.

### Applied implications

With our studies we aim at increasing the general awareness of linguistic factors, and specifically the use of established terms, when communicating about cases of sexual violence. Our results underline the existence and potential importance of differing self-perceptions and perceptions by others that are attached to specific labels. These differences should be considered in work supportive of women who were raped, in future research on sexual violence, and in media and everyday communication.

Practitioners working in contexts of social support and therapy, on the one hand, should be aware of the effects that using a specific label may have on their clients and, on the other hand, should consciously reflect the use of those labels in order to support the development of a differentiated and supportive self-concept (as recommended by [20], as well as [13]). An exclusive focus on the negative and stigmatizing aspects of a rape experience, which may be the result of using the label "victim" as a self-reference, may hinder the development of a "survivor" identity and an active and forward-looking coping with the experience. Researchers investigating the topic of sexual violence should not only develop an increased consciousness for the labels and their potential effects, but should also become aware of the impact that the use of a specific label may have when reporting empirical results. In everyday communication and the media, the use of a particular label may have massive effects on the public's perception of women who have experienced sexual violence and, more specifically, on the public's intention to support these women or organizations caring for them. If the "survivor" label highlights a woman's strength and forward-looking processing of the violence she experienced, using this label may also cause the woman's social environment to provide her less social support. Conversely, under certain circumstances (e.g. when there is little or no information provided on the actual sexual assault) the term can increase the estimation of a rape’s severity despite the positive connotations it carries and may therefore lead to increased social support provided. The associations of the "victim" label with weakness and passivity may contribute to the public stigmatization of women who were raped, and make it more difficult for them to adopt an optimistic perspective. In her dissertation, Hockett [26] reports first evidence that the terms "victim" and "survivor" may differentially affect participants' intention to help. Such effects should be investigated further in future research.

On the other hand, as shown in Studies 2 and 3, one must be aware that the term "survivor" cannot be regarded as generally advantageous and therefore to be endorsed in the communication of sexual violence. Whereas the label may be regarded as beneficial in the context of coping with experiences of sexual violence and when focusing on long-term consequences, it may also indicate a higher level of severity than may be warranted by the specifics of the case when used in a context that emphasizes more immediate outcomes. Furthermore, the term needs to be applied selectively as its use appears to be considered as inappropriate depending on the context it is applied to.

In all of the contexts mentioned, people should strive for a more conscious, differentiated, and respectful use of labels, as our studies as well demonstrate the complexity of experiencing and communicating about sexual violence and its outcomes. Specific recommendations for the labels' use in particular contexts as well as in specific languages will strongly depend on the results of future studies.
